# Evaluation of Substantia Nigra morphology in Parkinson’s Disease

**DOI:** 10.1097/MD.0000000000037538

**Published:** 2024-03-22

**Authors:** Nazlı Durmaz Çelik, Asli Yaman Kula, Uğur Toprak, Suzan Saylısoy, Aydan Topal, Serhat Özkan

**Affiliations:** a Department of Neurology, Eskişehir Osmangazi University Faculty of Medicine, Eskişehir, Turkey; b Department of Neurology, Bezmialem Foundation University Faculty of Medicine, Istanbul, Turkey; c Department of Radiology, Eskişehir Osmangazi University Faculty of Medicine, Eskişehir, Turkey.

**Keywords:** Parkinson disease, signal intensity, substantia nigra, volume asymmetry

## Abstract

In the elderly population, Parkinson’s Disease (PD) is the second most common neurodegenerative disorder and is associated with morphological changes in the basal ganglia, especially the substantia nigra (SN). This study aimed to evaluate the volume and signal intensity (SI) of SN using Magnetic Resonance Imaging (MRI) to detect structural changes and investigate the relationship between the onset side and disease severity of PD. Clinical features and imaging data of 58 patients with PD were retrospectively analyzed from their medical records. Axial T2-weighted fluid-attenuated inversion recovery (FLAIR) sequences of 3 Tesla (T) MRIs were used for the measurements. The right and left SN volumes and SI measurements were calculated in duplicate by 2 blinded and qualified neuroradiologists. The side of disease onset, disease duration, levodopa equivalent daily dose, Movement Disorder Society-sponsored Unified Parkinson Disease Rating Scale (MDS-UPDRS III) motor score, and modified Hoehn and Yahr (H&Y) scale scores were recorded and compared with SN volume and SI measurements. No statistically significant difference was found between the disease onset side and contralateral SN volume or SI measurements (*P* > .05). Despite high inter- and intra-rater reliability rates, there was no significant difference in the volume and SI of the contralateral SN according to H&Y stages (*P* > .05). Furthermore, SN volume and SI measurements were not significantly correlated with disease duration and MDS-UPDRS III motor score (*P* > .05). SN volume and SI values measured using axial FLAIR 3T MRI are not correlated with the side of onset or disease severity in PD. New imaging methods are required to detect preclinical or early-stage PD.

## 1. Introduction

Parkinson’s Disease (PD) is a neurodegenerative condition characterized by the degeneration of dopaminergic neurons in the substantia nigra (SN), a key region of the midbrain essential for the regulation of motor function.^[1,2]^ Anatomical studies have revealed that the SN comprises 2 distinct parts with disparate connections and functions: the pars compacta (SNpc) and the pars reticulata (SNpr). PD mainly causes the degeneration of dopaminergic neurons in the SNpc, resulting in reduced dopamine levels in the striatum, particularly in the caudal section and the head of the caudate nucleus.^[3,4]^ Although the clinical diagnosis of PD depends on the existence of motor symptoms, resting tremors, bradykinesia, and rigidity, the underlying pathological alterations commence years before the initiation of these symptoms.^[1]^ At least 70% to 80% of dopaminergic neurons in the SNpc are lost before the onset of the clinical symptoms of PD.^[5,6]^

Neuroimaging methods, such as Magnetic Resonance Imaging (MRI), have been pivotal in exploring structural and functional alterations within the SN in PD.^[7,8]^ The conjecture that iron deposition in the SN is linked to PD pathogenesis^[9]^ led to the assessment of iron content through MRI relaxometry in high-field strength units, revealing early increased deposition.^[10,11]^ Advanced MRI methods may establish exquisite alterations in the SN, which is crucial for diagnosing preclinical and early stages of PD and monitoring disease progression and treatment efficacy.^[12,13]^ However, the relationship between SN volume, signal intensity (SI), and motor asymmetry in PD is poorly understood. We aimed to investigate SN volume and SI as potential markers of motor asymmetry in PD using volume and SI analysis of axial T2-weighted Fluid-attenuated inversion recovery (FLAIR) 3 Tesla (T) MRI sections.

## 2. Methods

Patients admitted to the neurology outpatient clinic of Eskişehir Osmangazi University Medical Faculty Hospital between August 2021 and June 2022 were included in this study. The data of 58 patients with varying stages of PD were retrospectively evaluated from hospital records. All patients with PD met the diagnostic criteria outlined by the UK Parkinson Disease Brain Bank.^[14]^ The study protocol followed the principles of the Declaration of Helsinki and was approved by the local Institutional Review Board (No: 29 Date: 26.02.2019).

Patients lacking MRI scans or exhibiting abnormal MRI results; those with neurological or medical conditions affecting central nervous system function; those with a history of head trauma involving more than a few minutes of loss of consciousness, learning disabilities, and psychiatric conditions such as schizophrenia, personality disorder, bipolar disorder, or history of substance dependence were excluded. Anxiety and depression were not included in the exclusion criteria as they are commonly comorbid with PD. Clinical assessments included age, gender, disease duration, onset side of PD, levodopa equivalent daily dose (LEDD), Movement Disorders Society-sponsored revision of the Unified Parkinson Disease Rating Scale (MDS-UPDRS III) motor score, and modified Hoehn and Yahr (H&Y) scale score.

### 2.1. MRI protocol and image analysis

MRI data were obtained using a 3 T GE 750 W scanner (Siemens Healthcare, Erlangen, Germany) with a 32-channel head coil. Axial T2-weighted FLAIR sequences were used for SN volume^[15]^ and SI measurements. The imaging parameters were as follows: slice thickness, 2 mm; field of view, 22 cm; repetition time, 8075 ms; echo time, 100 ms; matrix of 320 × 224, and NEX, 1. The sections were arranged perpendicular to the floor of the fourth ventricle, with the median line of the slab placed at the center of the mesencephalon.

To measure the size of the SN, the right and left SN regions of interest (ROI) were measured by 2 qualified senior neuroradiologists (UT and SS, authors). They were blinded to the patients’ clinical information and performed measurements separately. The interval between the 2 measurements was at least 48 hours. Osirix Lite DICOM viewer was used for the measurements. The SN was identified on axial FLAIR sequences at the most significant magnification, which did not impair the image resolution. It was used using the established free ROI method criteria, and the boundaries of the ROI were manually drawn. The left and right SN were considered independently, and the volume and intensity were calculated twice for each patient by each neuroradiologist.

To ensure the reliability of the measurements, inter-observer and intra-observer variability was assessed by calculating the intraclass correlation coefficient for both the SN volumes and SI measurements.

### 2.2. Data analyses

Statistical analyses were performed using IBM SPSS 11.5 (SPSS Inc., Chicago, IL) software. For numerical variables with a normal distribution, mean ± standard deviation was used, while non-normally distributed numerical variables were presented as median (minimum-maximum). Categorical variables were expressed as frequencies (percentages). The independent sample t-test was used to assess the differences between the 2 measurements for normally distributed numerical variables. For 3 or more groups, comparisons were conducted using a one-way analysis of variance test if the data were normally distributed and the Kruskal-Wallis test otherwise. The relationships between the variables were determined using correlation analysis. Consistency between measurements was determined using the intraclass correlation coefficient. P values <0.05 were considered to indicate a significant difference.

## 3. Results

Of the 58 patients with PD, 24 (41.3%) were female. The mean age was 58.75 ± 8.68 years. The mean disease duration was 14.81 ± 5.85 years. The disease onset side was right in 22 (37.9%) patients, left in 31 (53.4%) patients, and bilateral in 5 (8.6%) patients. The mean MDS-UPDRS III motor score was 35.18 ± 97. The mean LEDD was 1097.91 ± 358.16 mg. H&Y scale score was 2.97 ± 0.7 (Table 1).

**Table 1:** Demographic and clinical data of patients.

Age (yr, mean ± SD)	58.75 ± 8.68
Gender	
Female (n,%)	24 (41.3%)
Male (n,%)	34 (58.7%)
Onset side	
Right (n,%)	22 (37.9%)
Left (n,%)	31 (53.4%)
Bilateral (n,%)	5 (8.6%)
Disease duration (yr, mean ± SD)	14.81 ± 5.85
LEDD (mg)	1097.91 ± 358.16
MDS-UPDRS III score (mean ± SD)	35.19 ± 7.97
Hoehn and Yahr scale score (mean ± SD)	2.97 ± 0.7

Disease duration refers to the time between the onset of symptoms and the time of the MRI.

LEDD = Levodopa equivalent daily dose, MDS-UPDRS = Movement Disorder Society-sponsored revision of the Unified Parkinson Disease Rating Scale, SD = standard deviation.

The inter-rater reliability was 98% (95% CI = 0.943–0.991) for the right SN volume measurement and 98.3% (95% CI = 0.967–0.991) for the left SN volume measurement. For rater A, the intra-rater reliability was 99.2% (95% CI = 0.987–0.995) for the right SN volume and 98.8% (95% CI = 0.979–0.993) for the left SN volume measurements. For rater B, the intra-rater reliability was 96.4% (95% CI = 0.939–0.979) for the right SN volume and 97.3% (95% CI = 0.954–0.984) for the left SN volume measurements (Table 2).

**Table 2:** Intraclass correlation coefficient of measurement with 95 percent confidence intervals for intra- and inter-rater reliability of substantia nigra volume.

Raters	Right substantia nigra volume		Left substantia nigra volume	
	Mean ± SD	ICC (95% CI)	Mean ± SD	ICC (95% CI)
A	65.03 ± 13.87	0.992 (0.987–0.995)	66.83 ± 15.09	0.988 (0.979–0.993)
B	67.10 ± 14.05	0.964 (0.939–0.979)	68.28 ± 14.16	0.973 (0.954–0.984)

Inter-rater reliability				
Raters	Right substantia nigra volume		Left substantia nigra volume	
	Mean ± SD	ICC (95% CI)	Mean ± SD	ICC (95% CI)
n = 2	66.07 ± 13.86	0.980 (0.943–0.991)	67.56 ± 14.52	0.983 (0.967–0.991)

ICC = intraclass correlation coefficient.

The inter-rater reliability was 96.3% (95% CI = 0.933–0.979) for the right SI and 97.2% (95% CI = 0.949–0.984) for the left SI measurements of the SN. For rater A, the intra-rater reliability was 99.8% (95% CI = 0.997–0.999) for the right SI and 99.8% (95% CI = 0.997–0.999) for the left SI measurements. For rater B, the intra-rater reliability was 98.8% (95% CI = 0.980–0.993) for the right SI and 99.3% (95% CI = 0.988–0.996) for the left SI measurements (Table 3).

**Table 3:** Intraclass correlation coefficient of measurement with 95% confidence intervals for intra- and inter-rater reliability of substantia nigra signal intensity.

Raters	Right substantia nigra intensity		Left substantia nigra intensity	
	Mean ± SD	ICC (95% CI)	Mean ± SD	ICC (95% CI)
A	889.47 ± 199.53	0.998 (0.997–0.999)	888.68 ± 185.21	0.998 (0.997–0.999)
B	866.35 ± 171.10	0.988 (0.980–0.993)	869.12 ± 171.85	0.993 (0.988–0.996)

Inter-rater reliability				
Raters	Right substantia nigra intensity		Left substantia nigra intensity	
	Mean ± SD	ICC (95% CI)	Mean ± SD	ICC (95% CI)
n = 2	877.91 ± 182.82	0.963 (0.933–0.979)	878.91 ± 176.44	0.972 (0.949–0.984)

ICC = intraclass correlation coefficient.

No statistically significant correlation was observed between the disease duration, MDS-UPDRS III motor score, and SN volume. There was a weak negative correlation between the LEDD and contralateral SN volume (Table 4). No statistically significant correlation was found between the disease duration, LEDD, MDS-UPDRS III motor score, and SI of the SN (Table 5).

**Table 4:** Spearman correlations between clinical data and Substantia Nigra volumes.

	Contralateral SN volume	Ipsilateral SN volume
Disease duration		
Correlation coefficient (r)	−0.223	−0.129
* P*	.109	.356
MDS-UPDRS III score		
Correlation coefficient (r)	0.193	0.210
*P*	.165	.131
LEDD		
Correlation coefficient (r)	−0.299	−0.221
* P*	**.030**	.112

LEDD = Levodopa equivalent daily dose, MDS-UPDRS = Movement Disorder Society-sponsored revision of the Unified Parkinson Disease Rating Scale, SN = substantia nigra.

**Table 5:** Spearman correlations between clinical data and Substantia Nigra signal intensity.

	Contralateral SN intensity	Ipsilateral SN intensity
Disease duration		
Correlation coefficient (r)	0.032	0.081
*P*	.821	.565
MDS-UPDRS III score		
Correlation coefficient (r)	0.050	0.100
*P*	.725	.477
LEDD		
Correlation coefficient (r)	−0.072	−0.110
*P*	.607	.433

LEDD = Levodopa equivalent daily dose, MDS-UPDRS = Movement Disorder Society-sponsored revision of the Unified Parkinson Disease Rating Scale, SN = substantia nigra.

No statistically significant difference was found between the disease onset side and the contralateral SN volume (for right onset side *P* = .637, for left onset side *P* = .789) or SI measurements (for right onset side *P* = .676, for left onset side *P* = .524). The contralateral SN volume and SI measurements did not differ according to H&Y stages (*P* = .055, *P* = .181 respectively). Similarly, no association was observed between the disease onset side and disease duration (*P* = .899) or between the H&Y stage and disease duration (*P* = .327).

## 4. Discussion

The SN is a pair of tilted plate-like structures within the antero-supero-lateral aspect to the postero-infero-medial aspect through the entire midbrain. Neurons pigmented by neuromelanin in the SNpc contain high dopamine concentrations and are known to be the principal source of striatal dopamine. On the other hand, neurons in SNpr comprise gamma-aminobutyric acid (GABA), which projects to the thalamus and pedunculopontine tegmental nucleus.^[16]^

Several studies have been conducted to identify the differences in SN volume between individuals with PD and controls using MRI, with varying results. Certain studies have observed no volumetric disparities in the SN compared with the control groups.^[17,18]^ In contrast, other studies described a decrease^[19]^ and even an increase in SN volume in patients with PD.^[20]^ Minati et al^[9]^ reported a characteristic lateral-medial loss of the SN with decreased volume in patients with PD, whereas Péran et al^[21]^ noted no volumetric changes. Kwon et al^[20]^ demonstrated that the ripple on the lateral surface of the SN of patients with PD was more prominent on the side, contrary to the symptoms that were more severe. However, SN volumes showed no laterality-based differences between the contralateral and ipsilateral sides relative to the more affected side. Similarly, in our study, the contralateral and ipsilateral SN volumes did not show a statistically significant difference according to the side of the disease onset. Geng et al^[17]^ showed no significant volumetric difference in the caudate or SN between early-stage PD, advanced-stage PD, and controls on T1-weighted images obtained using 3 T MRI. Vitali et al^[19]^ showed that volume measurement of the SN with T1-weighted 3 T MRI prepared with magnetization transfer differentiates between the PD stages. In 7 T MRI T2-weighted axial images, a significant difference was noted between normal and PD patients and between groups with different MDS-UPDRS III motor scores for PD in the distance measurement of the SN from the lateral and ventral and from the midline to the lateral border.^[22]^ In a study involving 29 patients with PD (H&Y stages 1–3), the basal forebrain was visualized using T2-weighted FLAIR images. Compared to controls, 13 patients with H&Y stage 1 PD showed significantly reduced SNpc volumes. A significant reduction in BF volume was observed in H&Y stage 2 or 3 PD patients compared to controls and H&Y stage 1 PD patients.^[15]^ When we examined the SN volumes according to the H&Y stage, there was no significant decrease in volume as the disease severity increased. In addition, there was no correlation between MDS-UPDRS III score, disease duration, and contralateral SN volumes.

Morphological changes, likely attributed to iron accumulation and degeneration in the SNpc, may cause enlarged hypointense fields on MRI. At lower resolutions, some authors have reported increased SN volumes, which may be explained by observed morphological alterations. These alterations are consistent with longitudinal reports of increased R2* relaxation rates, an indirect measure of the iron level in the deep gray matter, of the SN in PD (10.2% in pars compacta, 8.1% in pars reticulata). This increase can be attributed to ferritin-induced field inhomogeneities and has been demonstrated to correlate with the deterioration of motor symptoms.^[23]^ A recent study found a greater change in R2* relaxation rates in the SNpc of patients who were likely to experience freezing of gait early on in PD. However, the study was limited by the small sample size of only 19 participants, and these results need to be confirmed in a more extensive study.^[6]^ Péran et al^[21]^ identified an elevated R2* transverse relaxation rate in the SN of PD patients. Our study did not show a statistically significant difference between contralateral and ipsilateral SI according to the side of the disease onset. The degree of motor impairment and alterations in iron buildup in the SN of PD patients were not correlated, according to Graham et al^[24]^ However, according to Gorell et al,^[25]^ SN iron-dependent MRI contrast and simple response time, a gauge of motor function, are correlated. In this study, we did not observe any correlation between the MDS-UPDRS III score or disease duration and contralateral SI of the SN in PD patients. These findings suggest that lateralization or disease severity is not directly correlated with SN volume or SI.

A study compared the diagnostic accuracy of 3D FLAIR and dopamine transporter (DAT) imaging in 45 patients with Parkinsonism. The study defined intact nigrosome-1 as oval or linear hyperintensities on the posterolateral side of the SN. The results showed that 3D FLAIR imaging had a sensitivity of 85.7% and a specificity of 85.4% in identifying nigrosome-1 loss.^[26]^ Cho et al^[22]^ defined the term “smudging” to describe the loss of fine boundaries between the substantia nigra (SN) and crus cerebri, which appears relatively “serrated” in PD patients when observed through 7 T MRI. Additionally, a 3-layered anatomical organization of the SN was identified on susceptibility-weighted (SW) 7 T MRI in normal controls, which was less evident or unidentifiable in patients with PD. The architectural change derived from SW imaging in the substantia nigra allowed for perfect discrimination between PD patients and normal controls, achieving a sensitivity of 100% and specificity of 96.2%.^[27]^ Recent studies have shown that the proper anatomical position of the SN is in fast short inversion recovery (IR)-weighted images.^[18]^ Hutchinson and Raff^[28]^ and Minati et al^[9]^ demonstrated a notable difference in the SN area between controls and PD patients, measured on specific combinations of IR-weighted images. In our study, the lack of difference in volume measurements according to disease severity in PD patients and between ipsilateral and contralateral volume measurements may be due to the use of a less advanced imaging method such as 3 T MRI.

Our study had some limitations. First, we did not compare patients with PD with the controls. Therefore, we were unable to perform a direct comparison. The number of patients was limited, the existing patient groups were not homogeneous, and the number of patient groups included in H&Y stages 2 and 3 was relatively high compared with the number of patients in other stages. Also, there are imaging techniques that are more efficient than T2-weighted FLAIR sequences used to visualize SN, and 3 T MRI is a lower-strength MRI.

In conclusion, we found no statistically significant difference between symptom onset side and SN volume or SI measurements. We did not observe any correlation between these measurements and disease severity. Axial T2-weighted FLAIR sequences of 3 T MRI were preferred because they are easily accessible and widely used in hospitals. These findings are surprising given the reliability of our measurements, as structural changes in the SN have previously been proposed as potential biomarkers of PD.^[13]^ Intriguingly, structural changes in the SN in PD can be revealed by different imaging modalities, and it seems that in the future, it will be essential to determine which measurement better predicts the clinical conversion to PD. Therefore, new studies with more valid measurement methods are needed, perhaps in more early- or advanced-stage patient groups with more samples.

## Author contributions

**Conceptualization:** Nazli Durmaz Çelik, Serhat Özkan.

**Data curation:** Uğur Toprak, Suzan Saylisoy.

**Formal analysis:** Asli Yaman Kula, Aydan Topal.

**Investigation:** Asli Yaman Kula.

**Methodology:** Nazli Durmaz Çelik, Serhat Özkan.

**Supervision:** Serhat Özkan.

**Writing – original draft:** Nazli Durmaz Çelik, Serhat Özkan.

**Writing – review & editing:** Nazli Durmaz Çelik, Asli Yaman Kula.
